# Tracking the environmentally responsive intracellular fate and autophagic interactions of mesoporous silica nanoparticles via gap spectral detection

**DOI:** 10.3389/fbioe.2026.1961020

**Published:** 2026-09-22

**Authors:** Yuanyuan Yan, Yanmin Hu, Yuxuan Cheng, Jinli Sun, Xingjie Hu

**Affiliations:** 1 School of Public Health, Shanghai Jiao Tong University School of Medicine, Shanghai, China; 2 Department of Gerontology, The Second Affiliated Hospital of Zhengzhou University, Zhengzhou, Henan, China; 3 School of Clinical Medicine, Guizhou Medical University, Guiyang, Guizhou, China

**Keywords:** cellular autophagy, environmentally responsive biomaterials, gap spectral detection, live-cell imaging, mesoporous silica nanoparticles (MSNs), organelle interactome

## Abstract

Understanding the complex intracellular fate and autophagic mechanisms of environmentally responsive biomaterials is essential for advancing targeted disease therapies. However, monitoring these dynamic interactions in living cells is frequently hindered by the spectral crosstalk of traditional fluorescence microscopy and the requirement for specialized multichannel GaAsP/PMT detector arrays with high digitization depth. Herein, we develop a gap spectral detection method that captures spatial and spectral information across the visible range using a general confocal microscope. By implementing defined interval gaps, this approach eliminates excitation laser interference, enabling high-fidelity, six-color live-cell 2D and 3D imaging. We utilized this method to systematically track the intracellular routing of mesoporous silica nanoparticles (MSNs), which utilize the acidic pH of lysosomes as an environmental trigger for the proton sponge effect. Our analysis revealed that MSNs induced cellular autophagy and dynamically altered the organelle interactome. Notably, following the proton sponge effect, MSNs escaped from lysosomes after 6 h and subsequently increased their co-localization with the Golgi apparatus and endoplasmic reticulum. These findings demonstrate that our gap spectral imaging technique provides an accessible and ultra-multiplexed analytical tool for evaluating the intracellular fate of responsive biomaterials, thereby facilitating the rational design of nanomedicines.

## 1 Introduction

Mesoporous silica nanoparticles (MSNs) have emerged as highly promising environmentally responsive biomaterials for the treatment of major diseases, particularly in targeted drug delivery and tumor therapy (Chen et al., 2018; Ding et al., 2023; Li et al., 2021; Wang et al., 2018). Thanks to their tunable pore sizes, large internal surface area, and easily functionalized exterior surfaces, MSNs serve as exceptionally versatile carriers capable of encapsulating diverse therapeutic payloads, including small-molecule drugs, nucleic acids, and proteins. Upon cellular internalization, these nanocarriers navigate complex intracellular environments, often utilizing mechanisms such as the proton sponge effect-triggered by the acidic pH of lysosomes-to escape acidic lysosomes and exert their therapeutic functions (Foroozandeh and Aziz, 2018; Hua and Wang, 2022; Huai et al., 2019; Liang et al., 2023; Tao et al., 2025). During this process, MSNs inevitably interact with various subcellular compartments and frequently trigger cellular autophagy, a critical biological response closely associated with disease progression and the ultimate therapeutic efficacy of the biomaterials (Huang et al., 2010; Li et al., 2012; Li et al., 2019; Lin and Haynes, 2010; Lu et al., 2009).

To rationally design and evaluate these responsive biomaterials, it is imperative to systematically monitor their dynamic intracellular fate and real-time interactions with the organelle interactome (Bai et al., 2026; Park et al., 2026; Prashar and Puertollano, 2026; Valm et al., 2017; van Tartwijk et al., 2026). Fluorescence microscopy is widely employed for such noninvasive cellular tracking (Hu et al., 2023; Xie et al., 2020). However, the broad emission spectra of conventional fluorophores typically limit simultaneous imaging to only three or four targets within the visible range (Garini et al., 2006; Ikonen, 2008; Neher and Neher, 2004). Introducing more fluorescent markers inevitably leads to severe spectral crosstalk, which obscures the precise spatial relationships between the biomaterials and organelles (Tao et al., 2025; Wang et al., 2025; Zhang et al., 2025). While ultra-multiplexed spectral imaging using multichannel GaAsP/PMT detector arrays with high digitization depth or white lasers with gating functions can effectively resolve this crosstalk without excitation laser interference, these specialized instruments are prohibitively expensive and largely inaccessible to many general laboratories (Valm et al., 2017).

To bridge this technological gap and facilitate the broad evaluation of biomaterials, we propose a gap spectral imaging method utilizing a standard general confocal microscope. By operating four common excitation lasers (405 nm, 488 nm, 552 nm, and 638 nm) in spectral acquisition mode, we sequentially acquire visible range spectra in defined bands, strategically setting interval gaps to eliminate excitation light interference. This straightforward approach enables crosstalk-free six-color 2D and 3D live-cell imaging without signal loss. In this study, we applied this method to map the intricate organelle interactome during MSN-triggered autophagy, providing quantitative insights into the spatiotemporal intracellular routing of these responsive nanocarriers.

## 2 Materials and methods

### 2.1 Cell culture and plasmid transfection

HeLa cells were cultured in Minimum Essential Medium (MEM) supplemented with 10% heat-inactivated fetal bovine serum (FBS) and antibiotics (100 μg/mL of streptomycin and 100 U/mL of penicillin). The cells were maintained in a humidified incubator at 37 °C with 5% CO2. For live-cell confocal imaging, cells were seeded in glass-bottom confocal dishes. To visualize specific subcellular organelles, cells were transiently transfected with specific fluorescent fusion protein plasmids: pmTurquoise2-Golgi (Addgene #36205) for the Golgi apparatus, LAMP1-mRFP (Addgene #1817) for lysosomes, a Sec61 beta-YFP fusion construct (incorporating the bovine prolactin signal sequence) for the endoplasmic reticulum (ER), and a mitochondria-targeting signal fused to mPLUM for mitochondria. Transfections were carried out utilizing the Lipofectamine 3000 reagent (Invitrogen) according to the manufacturer’s recommended protocols, with 200 ng of each respective plasmid administered per dish.

### 2.2 Preparation and fluorescent labeling of mesoporous silica nanoparticles (MSNs)

Environmentally responsive mesoporous silica nanoparticles (MSNs) were synthesized via a modified sol-gel precipitation protocol. Briefly, hexadecyltrimethylammonium bromide (CTAB, 1.82 g) and NH4F (0.5 g) were dissolved and continuously stirred in an 80 °C oil bath for 1 h. Subsequently, 6 mL of tetraethyl orthosilicate (TEOS) was added dropwise to the mixture to initiate the hydrolysis and condensation reactions, which were allowed to proceed for 2 h. The resulting MSNs were isolated by centrifugation (10,000 rpm for 15 min) and washed thoroughly with deionized water and absolute ethanol to remove residual reactants. For fluorescent tracking, the purified MSNs were dispersed in phosphate-buffered saline (PBS) and conjugated with Atto 647-NHS ester. The labeling reaction was performed under gentle stirring at room temperature for 2 h. The finalized MSN-Atto647 nanoparticles were harvested via centrifugation (10,000 rpm for 15 min) and repeatedly washed with PBS to eliminate unreacted dye.

### 2.3 Gap spectral image acquisition and linear unmixing

Live-cell imaging was conducted utilizing a Leica TCS SP8 confocal laser scanning microscope equipped with a dedicated live-cell environmental incubation chamber and an HC PL APO 63×/1.4 NA oil-immersion objective. To implement the gap spectral detection method, multiple fluorophores were excited using four standard diode lasers (405, 488, 552, and 638 nm) paired with a 488/552/638 nm main beam splitter. The excitation was conducted in a semi-interleaved/line-switching manner to avoid photobleaching and detector saturation. Fluorescence emission signals were captured utilizing photomultiplier tube (PMT) detectors operating in lambda mode. The spectral acquisition was configured to collect data in 10 nm bandwidth intervals (bins) across a detection range from 468 to 697 nm, with strategic gaps integrated to circumvent excitation laser backscatter. To establish accurate spectral reference profiles for linear unmixing, cells expressing single fluorophores were individually imaged under identical optical configurations, ensuring consistent laser power and master gain settings. The acquired multiplexed images were then processed using LAS AF software (Leica) to execute linear unmixing, effectively eliminating spectral crosstalk.

### 2.4 Image analysis and statistics

Following spectral unmixing, the resulting high-fidelity fluorescence images were segmented to delineate specific subcellular structures. Quantitative analysis of nanoparticle-organelle co-localization and inter-organelle contacts (interactome dynamics) was performed using the analytical modules within LAS AF software and ImageJ (FIJI).

## 3 Results

### 3.1 Establishment of the gap spectral detection method for multiplexed imaging

To precisely monitor the dynamic intracellular behavior of environmentally responsive biomaterials without relying on costly 32-bit spectral detectors or white lasers, we first established a robust gap spectral detection method on a conventional confocal microscope (Figure 1A). Standard sequential imaging modes are typically restricted to resolving a maximum of four fluorophores due to severe emission spectrum overlap. To overcome this limitation, our approach acquires fluorescent signals sequentially in distinct, narrow bands across the entire visible emission spectrum, deliberately setting intervals (gaps) between these bands to completely eliminate excitation light interference. Utilizing a standard laser configuration (405, 488, 552, and 638 nm), we systematically optimized the detection parameters. It was determined that a 10 nm spectral acquisition band coupled with a 20 nm gap provided the optimal signal-to-noise ratio. It is worth noting that these specific gap and band parameters are not universally fixed; they must be empirically customized when switching to a fundamentally different combination of fluorophores to balance signal collection efficiency and crosstalk avoidance. Because the band length and gap interval remain constant during the acquisition cycle, excitation light interference is strictly confined to within 3 nm of the spectral edges. To validate the accuracy of this acquisition strategy, we utilized HeLa cells expressing LC3-EGFP, co-transfected with four organelle markers (Golgi-pmTurquoise2, ER-EYFP, Lamp1-mRFP, and Mito-mPLUM), and incubated with Atto647-labeled MSNs. The fluorescence emission spectra extracted from these singly or multiply labeled samples using the gap spectral detection method perfectly matched the standard reference spectra for each respective fluorophore. Notably, no excitation laser bleed-through was observed in the sequentially collected images (Figures 1B,C), demonstrating that this method is highly capable of supporting ultra-multiplexed live-cell imaging.

**FIGURE 1:**
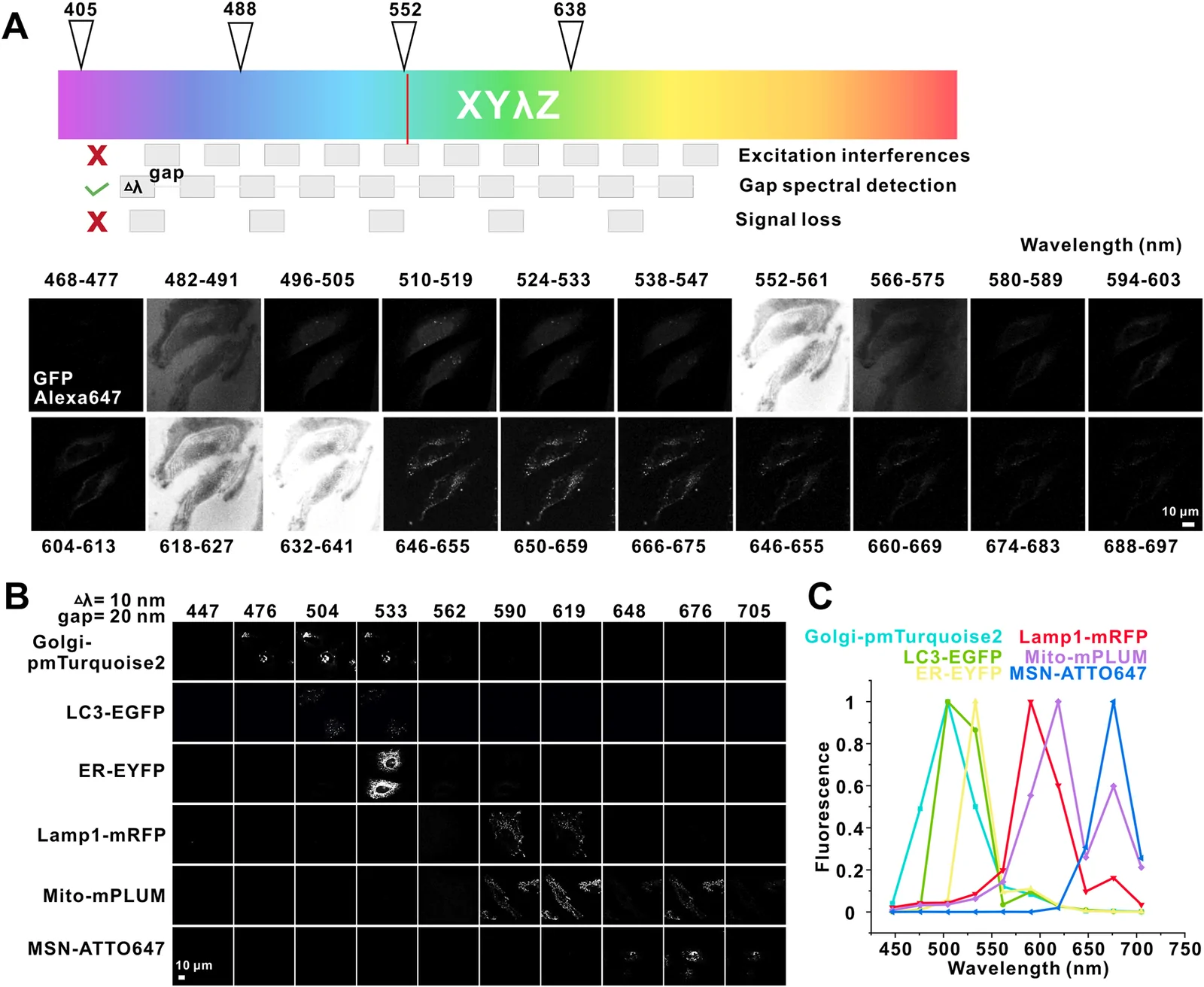
Principle and establishment of the gap spectral detection method. **(A)** Schematic illustration of the gap spectral detection strategy for eliminating excitation light interference. Unlike conventional continuous spectral acquisition, this method optimizes signal acquisition density and position by introducing specific intervals (gaps) to bypass the excitation laser wavelengths (405, 488, 552, and 638 nm). **(B)** Representative images of singly labeled cells acquired at specific wavelength bands. These images were captured under identical acquisition settings to establish the reference matrix for linear unmixing. **(C)** The corresponding emission spectra of the six distinct fluorophores derived from the singly labeled samples.

### 3.2 Validation of crosstalk-free ultra-multiplexed imaging in living cells

We subsequently evaluated the unmixing fidelity of the gap spectral detection method to ensure no loss of biological signals occurred during the imaging of complex systems. Initially, two fluorophores with slightly overlapping emission spectra were imaged (Figure 2A). Compared with traditional channel sequence imaging, the gap spectral detection method exhibited no discernible loss of fluorescence intensity in living cells, confirming the reliability of the signal acquisition process (Figures 2B,C). We then challenged the system with a highly overlapping fluorescent pair, EGFP and EYFP. These two fluorophores present a severe spectral unmixing challenge because their emission peak wavelengths are separated by only ∼20 nm, resulting in substantial spectral tail overlap that conventional channel sequence imaging fails to separate. (Figure 2D). As shown in the magnified region of Figure 2E, conventional sequential imaging failed to distinguish the signals, resulting in erroneous localization of LC3-EGFP puncta within the ER-EYFP channel. However, following the application of gap spectral detection and subsequent linear unmixing, the signals from the two proteins were perfectly separated without any loss of the true EGFP signal (Figures 2E,F). Having confirmed the unmixing efficiency, we applied this method to a highly complex, six-color live-cell model. HeLa cells stably expressing EGFP-LC3, transiently expressing markers for the Golgi, ER, lysosomes, and mitochondria, were treated with MSN-Atto647 for 12 h to trigger cellular autophagy. Utilizing gap spectral detection combined with linear unmixing via LAS AF software, we successfully achieved crosstalk-free, simultaneous imaging of all six fluorescent targets (Figure 3A). Furthermore, by executing this spectral imaging across consecutive XY planes with a 300 nm Z-axis step size (totaling 20 layers), we successfully rendered high-resolution 3D models of the multi-labeled cells. These results compellingly demonstrate that three-dimensional, ultra-multiplexed live-cell imaging can be readily achieved on general laboratory microscopes (Figure 3B).

**FIGURE 2:**
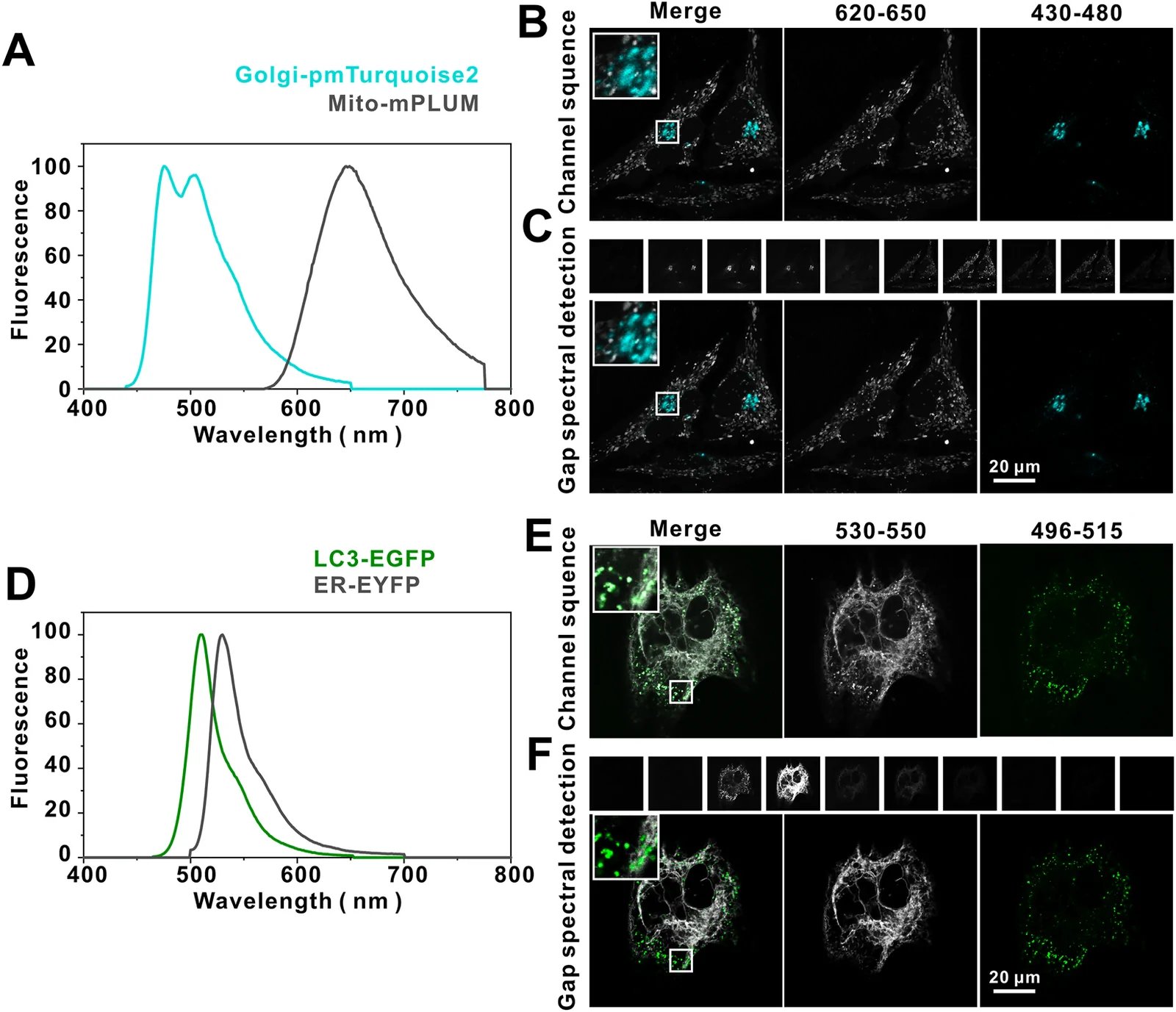
Validation of the gap spectral detection method for crosstalk-free multiplexed imaging. **(A)** Emission spectra of Golgi-pmTurquoise2 and Mito-mPLUM, exhibiting minimal spectral overlap. **(B)** Images acquired using the conventional channel sequence method. **(C)** Images acquired using the proposed gap spectral detection method followed by linear unmixing, confirming accurate signal separation without fluorescence loss. Single-label controls were generated using excitation laser lines at 405 nm for Golgi-pmTurquoise2, 488 nm for LC3-EGFP and ER-EYFP, 552 nm for LAMP1-mEFP, and 638 nm for Mito-mPLUM and MSN-Atto647. **(D)** Emission spectra of LC3-EGFP and ER-EYFP, showing severe spectral crosstalk. **(E)** Conventional sequential imaging fails to resolve the overlap, resulting in false co-localization (crosstalk) as indicated in the magnified region. **(F)** Successful signal separation of LC3-EGFP and ER-EYFP, achieved via gap spectral detection and linear unmixing.

**FIGURE 3:**
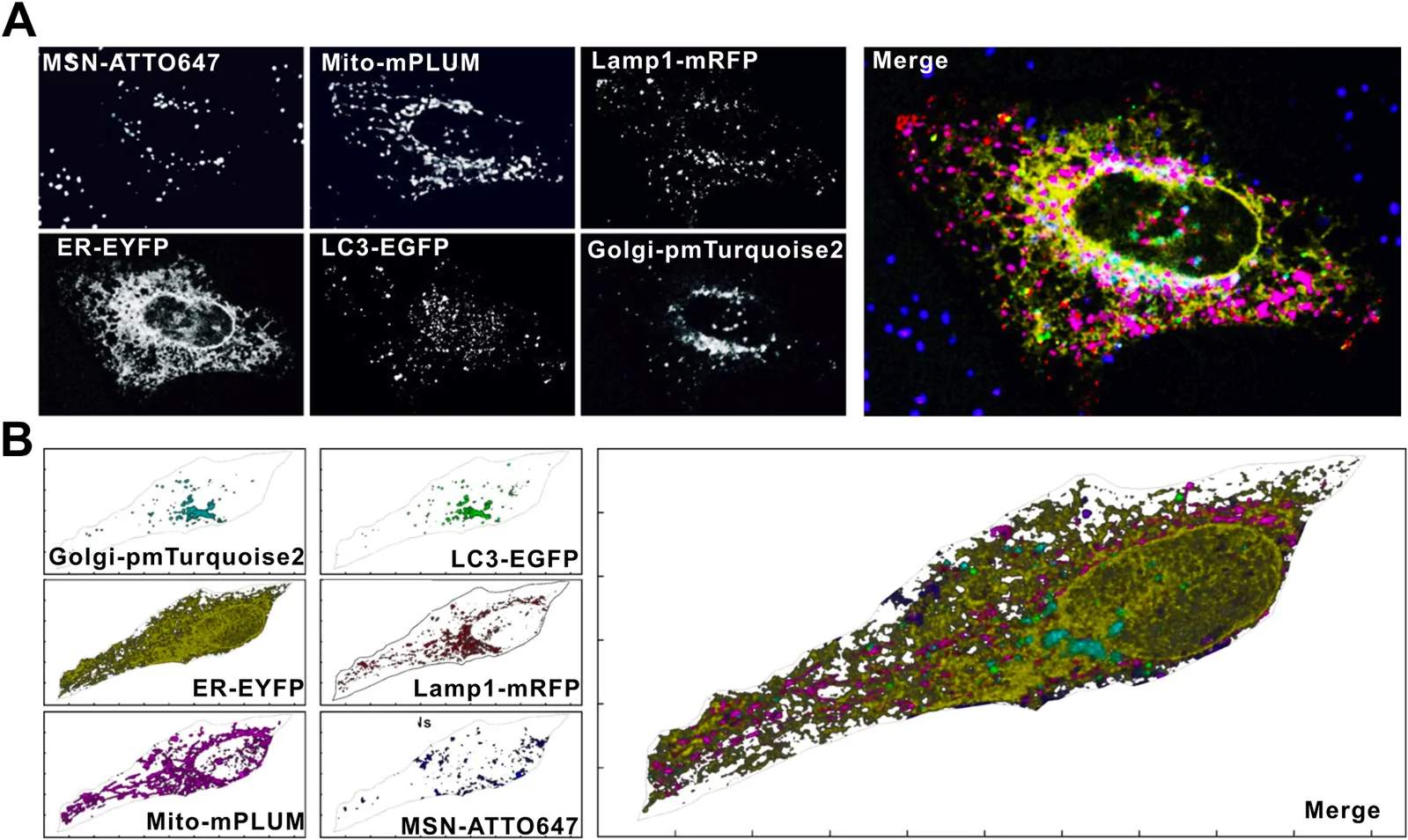
Six-color ultra-multiplexed live-cell imaging for tracking MSNs. HeLa cells stably expressing EGFP-LC3 were co-transfected with markers for the Golgi (pmTurquoise2), ER (EYFP), lysosomes (mRFP), and mitochondria (mPLUM), followed by incubation with Atto647-labeled MSNs for 12 h. **(A)** 2D and **(B)** 3D high-resolution micrographs of the multi-labeled living cell. Images were acquired utilizing the gap spectral detection method and processed via linear unmixing and volume rendering using LAS AF software, demonstrating precise spatial mapping of the nanocarriers and organelles.

### 3.3 Spatiotemporal mapping of the MSN-induced autophagic interactome

The intracellular routing of nanomaterials is a highly dynamic process that directly influences cellular stress responses and therapeutic outcomes (Guo et al., 2025; Ma et al., 2025; Ivleva et al., 2026; Rosero et al., 2026; Zhang et al., 2026; Zheng et al., 2026). The entry of MSNs into cells is known to trigger cellular autophagy, the extent of which escalates with increased nanoparticle uptake (Anderson and Simon, 2020; Behzadi et al., 2017; Jalouli et al., 2026; Ma et al., 2011; Studer et al., 2010). To map this complex biological response, we utilized the gap spectral imaging method to quantitatively analyze spatiotemporal alterations in the organelle interactome during MSN internalization (Figure 4A). By collecting and unmixing multiparametric images at various time points over 12 h, we tracked the dynamic intracellular fate of the environmentally responsive MSNs (Figure 4B). Quantitative co-localization analysis revealed that following endocytosis, the strongly positive-charged MSNs primarily accumulated within lysosomes during the initial 6 h. Strikingly, at 12 h post-incubation, the co-localization frequency between MSNs and lysosomes decreased from 47.6 (at 6 h) to 33.6, while their co-localization with the Golgi apparatus increased substantially. This phenomenon suggests that the MSNs induced a proton sponge effect within the acidic lysosomal microenvironment, leading to endosomal escape. Moreover, following this lysosomal escape, we observed an enhanced contact frequency between the Golgi and the endoplasmic reticulum (ER). These altered interactome dynamics strongly suggest the activation of a compensatory cellular clearance mechanism, wherein the host cells attempt to package and exocytose the escaped MSNs via the classical ER-Golgi secretory pathway.

**FIGURE 4:**
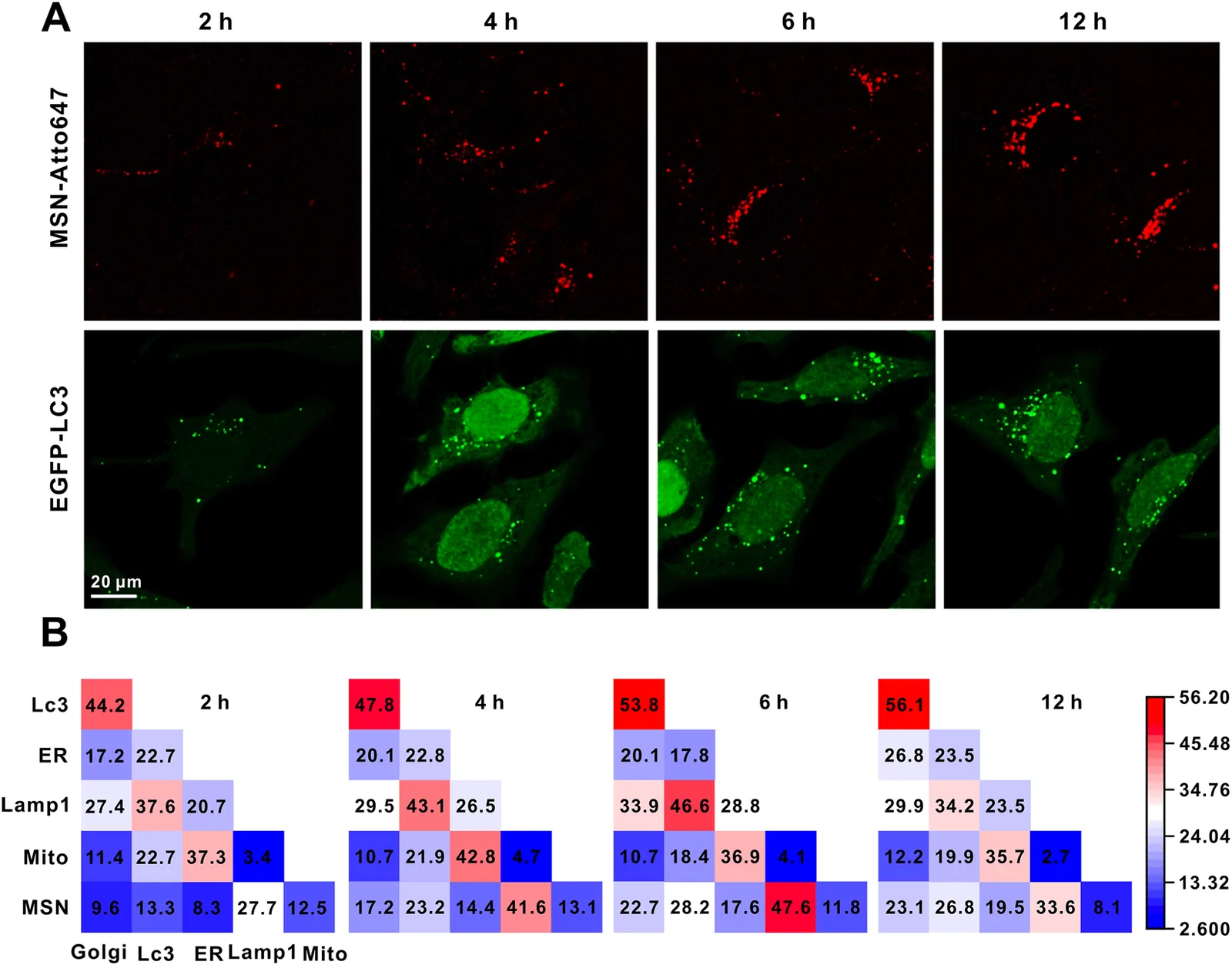
Spatiotemporal mapping of the organelle interactome and autophagic response triggered by MSNs. **(A)** Time-course confocal micrographs of Hela/EGFP-LC3 cells incubated with MSN-Atto647. The dynamic intracellular routing of MSNs and the progression of cellular autophagy were monitored at 2, 4, 6, and 12 h post-incubation. **(B)** Quantitative organelle interactome matrix displaying the spatiotemporal evolution of co-localization frequencies. The color-coded heatmap indicates the interactions between MSNs and various organelles over time, highlighting the environmentally responsive endosomal escape of MSNs and the subsequent enhancement of ER-Golgi contacts (*n* = 20 cells per time point).

## 4 Discussion

The rational design of environmentally responsive biomaterials for major disease treatment necessitates a comprehensive understanding of their dynamic intracellular routing and interactions. While spectral splitting imaging offers a window into these multifactorial biological mechanisms, its reliance on expensive 32-bit detectors or white lasers with gating functions has limited its widespread application. By implementing a gap spectral detection strategy on a conventional confocal microscope, our study provides an accessible and robust analytical platform for the simultaneous observation of ultra-multiple targets in living cells, enabling both 2D and 3D assessments of nanomedicine behavior.

Crucially, utilizing this multiplexed imaging approach, we mapped the intracellular interactome in response to MSN treatment. Our findings indicate that positively charged MSNs actively respond to the acidic lysosomal compartment, successfully executing endosomal escape via the proton sponge effect. Furthermore, the observation that escaped MSNs increasingly interact with the Golgi apparatus and ER highlights a complex cellular autophagic and clearance response. The enhancement of ER-Golgi contacts implies an active engagement of the exocytosis pathway, potentially as a mechanism to alleviate nanoparticle-induced cellular stress by packaging and clearing the foreign materials. Understanding this specific interactome, particularly how nanomaterials transit from lysosomes to the ER-Golgi network, provides critical insights for designing better environmentally responsive drug delivery systems. For example, nanocarriers can be engineered with precise surface modifications tailored to either accelerate lysosomal escape or exploit ER-targeted pathways to enhance intracellular payload release for tumor therapy.

Compared to relying solely on computational spectral unmixing algorithms, our gap spectral method offers hardware-based reliability by physically eliminating excitation interference during the acquisition phase. This preserves raw signal integrity before any mathematical processing is applied. Although our current methodology is constrained by a slight reduction in signal-to-noise ratio following linear unmixing, future optimizations utilizing vector-based unmixing algorithms may further enhance the detection sensitivity. Overall, this study not only presents a versatile imaging methodology but also provides valuable biological insights into the intracellular fate of responsive biomaterials.

## Data Availability

The raw data supporting the conclusions of this article will be made available by the authors, without undue reservation.
